# An Appeal to Every Registered Physician and Licensed Midwife in the United States for Information Concerning Criminal Abortion

**Published:** 1898-01

**Authors:** C. D. Arnold

**Affiliations:** El Reno, Okla.


### AN APPEAL TO EVERY REGISTERED PHYSICIAN AND LICENSED MIDWIFE IN THE UNITED STATES FOR INFORMATION CONCERNING CRIMINAL ABORTION.

DEAR DOCTOR: I most earnestly appeal professionally to each of you, regardless of your school of practice, your prominence in the medical profession, or your location, to answer the questions given below. In replying please designate each question by its number. Answers can be made in numerals, and if you do not elect to respond by letter a postal card will do as well. The face of such a card will present only an aggregation of meaningless figures to all who handle it except ourselves.

However, I will highly appreciate whatever you may impart in relation to criminal abortion otherwise than may be contained in your answers to my questions. I trust your visiting list, your cash and account books, and other data in your possession, will enable you to give *definite* or *approximate* answers without consuming too much of your time. If the 115,000 to 120,000 physicians in the United States will kindly give the information I ask, I will return to them through the medical press, some time during 1898, a summary of the results of my investigation.

I desire to assure you that every line given me on the subject of my inquiries will be held strictly private, if you request it, and should you not request its privacy, I will give it good treatment. If for any reason you wish to withhold your full name your initials will suffice. Remember my inquiries cover the year 1897, and where you can not give a *definite* answer an *approximate* answer is desirable.

### QUESTIONS.

1. Give total number of abortions from all causes that occurred in your practice during 1897.*

2. In how many of these abortions were the elements of criminality, to your mind, apparent?

3. In how many of these abortions, except those classed in question 2, were the elements of criminality, to your mind, probable?

4. How many of the abortions named in questions 2 and 3 were followed by puerperal septicæmia or other diseases?

---

* NOTE.—Question 1 should include abortions which you know occurred among your lady patrons without the attention of a reputable physician. Any abortion that resulted from an obstinate disregard on the part of the woman, of a physician's advice, or from the willful commission of any act which her observation, experience and other knowledge gave her reason to believe might induce immediately or even remotely the expulsion of the uterine contents, was criminal. (Any act, however simple, occurring in the daily avocation of a pregnant woman, if impelled by an intent, or even a desire or wish to get rid of pregnancy is criminal whether she aborts or not.) I use the word "abortion" here to mean the expulsion of the products of conception at any time during gestation to the end of the seventh month if the abortion was unavoidable, and to full term if criminal.

5. How many deaths resulted from abortions named in questions 2 and 3?

6. How many still-born in your practice?

7. How many infanticides?

8. How many viable children born in your practice?

9. How many cases of puerperal mania resulted from the abortions classed in questions 2 and 3?

All midwives who are licensed are solicited and urged to answer the above questions so far as their knowledge enables them. Doctor, permit me again to beg that you answer my inquiries either *definitely* or *approximately*, and if for any reason you can not fully answer all, do your best on questions two, three, five and nine. Medical journals throughout the United States are requested to favor the undersigned with an insertion of these questions in their January or February (1898) issues.

C. D. ARNOLD, M. D.

El Reno, Okla.

